# LAMP1 as a novel molecular biomarker to predict the prognosis of the children with autism spectrum disorder using bioinformatics approaches

**DOI:** 10.1038/s41598-023-40617-4

**Published:** 2023-08-28

**Authors:** Sisi Deng, Xiang Feng, Miao Yang, Wenjing Yu, Zixuan Wu, Xu Zhu, Zhenyan Song, Shaowu Cheng

**Affiliations:** College of Integrated Traditional Chinese and Western Medicine, Key Laboratory of Hunan Province for Integrated Traditional Chinese and Western Medicine on Prevention and Treatment of Cardio-Cerebral Diseases, Hunan University of Chinese Medicine, Changsha, 410128 China

**Keywords:** Biomarkers, Autism spectrum disorders

## Abstract

Autism spectrum disorder (ASD) is a neurodevelopmental disorder that usually manifests in childhood and is thought to be caused by a complex interaction of genetic, environmental, and immune factors. The majority of current ASD diagnostic methods rely on subjective behavioral observation and scale assessment, making early detection difficult. In this study, we confirmed that lysosomal-associated membrane protein 1 (*LAMP*1), a functional marker of immune cell activation and cytotoxic degranulation, was upregulated in ASD blood, brain cortex, and various genetic animal models or cells using bioinformatics approaches. The prognostic value of *LAMP*1 was investigated by correlating its expression with clinical ASD rating scales, and the receiver operating characteristic (ROC) curve analysis in ASD also revealed that it has a favorable diagnostic ability in distinguishing ASD from control cohort. According to gene set enrichment analysis (GSEA) results, LAMP1 correlated with genes that were enriched in natural kill and T cell immune function. Taking all of the evidence into account, we discovered that abnormal elevations of *LAMP*1 mRNA and protein in the blood of ASD children, may influence the development of ASD through its involvement in immune cell activity regulation. This report highlights a novel marker for ASD early detection as well as potential therapeutic targets.

## Introduction

Autism spectrum disorder (ASD) is a neurodevelopmental disorder characterized by impairments in social interaction, communication and restrictive and repetitive behaviors (RRB), and narrowed interests. ASD is considered to be the result of complex interactions among genetic, environmental, and immunological factors^1^. As the disease progresses, ASD will severely affect the quality of life of children in adolescence and adulthood, imposing a heavy economic burden on individuals, families and whole society.

Children with ASD are progressively more symptomatic by 12–24 months of age, but are not easily diagnosed^2^. While the clinical presentations of ASD are broad and varies significantly among individuals. Most current diagnostic methods for ASD are based on presenting behavioral criteria, but due to the subjective nature of presenting behaviors, most diagnostic methods and screening tools used for ASD are difficult to accurately assess in young children^3, 4^. Early diagnosis is critical for patients with ASD, as early intervention usually means better intervention outcomes^5^.

Biomarker research in ASD appears to be promising. Biomarkers that stratify ASD risk during the prenatal and postnatal pre-symptomatic periods may be especially useful for starting interventions early. Biomarkers that predict treatment response may speed up habilitation for those who have already been diagnosed^6^.

In this study, we first used bioinformatics methods to identify the LAMP1 (lysosome-associated membrane protein 1) as a potential candidate biomarker in ASD and further investigated its clinical correlation in autistic children. Similar to the previous study^7, 8^, we also found that LAMP1 had a function in regulating immune cell activity. Meanwhile, immune cell activity disorder has been reported in autistic children^9, 10^. Thus, we considered LAMP1 to be a valuable biomarker for ASD associated with immune cell dysregulation, capable of predicting the risk of ASD and assisting in early diagnosis, with significant clinical significance and application prospects.

## Materials and methods

### Data information

The expression matrix of four independent autism and control blood sample microarrays (GSE18123, GSE6575, GSE111176 and GSE87847) were retrieved in the GEO (Gene Expression Omnibus) database (https://www.ncbi.nlm.nih.gov/geo/). GSE18123 and GSE6575 were both the GPL570 Platform ([HG-U133 Plus 2]). GSE111176 used the GPL10558 Illumina HumanHT-12 V4.0 expression beadchip as the platform for Affymetrix Human, while GSE87847 used the GPL18281 Illumina HumanHT-12 WG-DASL V4.0 R2 expression bead chip. Furthermore, an RNA-seq dataset GSE212645 was selected as a validation data set to compare the blood expression levels of DEGs in ASD and healthy control. We also selected two expression datasets for autism brain tissues (GSE38322 and GSE28521) to validate the expression difference in brain. Four expression datasets from autistic mouse models (GSE81501, GSE72149, GSE50225 and GSE47150) were used to verify mouse *Lamp*1 gene expressions. GSE109905 dataset was used to analyzed the methylation alteration of CpG islands around the promoter region of human *LAMP*1 gene in the autistic children and control group.

### Differential gene expression analysis

After normalization of the raw microarray datasets downloaded, we used the limma package (version: 3.15) for microarrays and DESeq2 (version: 1.38.3) for RNA-seq datasets in R statistic software to obtain differential genes for each microarray, and then a combinatorial sorting analysis of these differential genes was performed using R language to obtain pooled differential genes. Differential expression profile of GSE212645 was downloaded in its supplementary table.

### Differential methylation analysis

We used the dataset GSE109905, a genome-wide DNA methylation profiling of ASD (n = 38) and controls (n = 31) using peripheral blood samples based on the platform GPL13534, the Illumina HumanMethylation450 BeadChip. The beta values were normalized by Beta Mixture Quantile Dilation (BMIQ) method. The limma package was used to analyze the differentially methylated positions (DMPs). The ratio of intensities between methylated and unmethylated alleles (beta-values) is used to assess methylation levels.

### Enrichment analysis

The Gene Set enrichment analysis (GSEA) of the Gene Ontology (GO) and Kyoto Encyclopedia of Genes and Genomes (KEGG) pathways for DEGs of interest and significant module was performed using the clusterProfiler package (version 3.16.1) in R statistical software. The Wikipathways enrichment analysis was performed in STRING (https://string-db.org/).

### Protein–protein interaction network construction

The protein–protein interaction networks (PPIs) of target proteins were built using the STRING (version 11.5) database (https://string-db.org/). The active interaction sources include text-mining, experiments, databases, co‑expression, neighborhood, gene fusion and co‑occurrence. The minimum required interaction score is 0.700 (high confidence). The network was subsequently visualized using the Cytoscape software (3.7.2) and the top 10 hub gene in the PPI was calculated using the cytoHubba plugin in Cytoscape using the Maximal Clique Centrality (MCC) algorithm.

### Research object recruitment

Children (n = 27) clinically diagnosed with ASD according to DSM-5 (Diagnostic and Statistical Manual of Mental Disorders, Fifth Edition) were recruited as study subjects from August 2020 to August 2021 at the First Hospital of Hunan University of Traditional Chinese Medicine (Changsha, Hunan Province, China). Normal control children (n = 15) were matched to the experimental group in terms of gender, age, nutritional status, family conditions, pregnancy history and family history. Autistic children (24 boys/3 girls) have an average age of 3.85 ± 0.77, while 15 healthy local children (11 boys/4 girls) with an average age of 4.40 ± 1.12. The descriptive statistics is shown in Supplemental Table 1. Subjects were excluded from somatic congenital genetic disorders or related psychosomatic disorders. The study was approved by the Ethics Committee of the First Hospital of Hunan University of Traditional Chinese Medicine (approval number: HN-LL-KY-2020-020-01). All parents of enrolled children or their guardians were fully informed and their informed consent was obtained before further experiments were performed.

### Peripheral blood mRNA extraction and reverse transcription

All subjects were enrolled and approximately 2 ml of peripheral venous whole blood was drawn into an empty vacuum tube containing EDTA (Ethylenediaminetetraacetic acid). Total RNA was extracted from the whole blood with TRIzol^®^ reagent (Invitrogen, USA) within one week. All mRNA was subjected to retro-transcribed to cDNA using a PrimeScript^®^ RT Master Mix Perfect Real Time kit (Takara Bio Inc.) according to the manufacturers' protocol.

### Droplet digital PCR

For gene expression quantification (copies/μL), we mixed the reaction buffer contained 1 × of QX200 ddPCR EvaGreen Supermix, 200 nM primers and 10 ng of template cDNA. For droplet generation, a total of 20 μL of reaction mixture and 70 μL of Droplet Generation Oil for EvaGreen were loaded in the Droplet Generation Cartridge, which was placed into the Droplet generator. 40 μL total droplet from each PCR reaction mixture were transferred to a 96-well PCR plate, which was sealed with a foil heat using PX1 PCR plate sealer. Amplification was performed in a C1000 Touch Thermal Cycler. Droplets were read with a QX200 Droplet Reader and analyzed using QuantaSoft software (Bio-Rad). More than 10,000 droplets were obtained and normalized to the appropriate housekeeping gene GAPDH (Glyceraldehyde 3-phosphate dehydrogenase). Values were reported as cDNA gene units per cDNA housekeeping units. All equipment and reagents were purchased from Bio-Rad (Bio-Rad Laboratories, Inc.). The sequence of primers is shown in Supplemental Table 2. Primers were synthesized by Sangon (Sangon Biotech Co., Ltd.).

### Phenotype prediction of LAMP1

We used the Harmonizome database (https://maayanlab.cloud/Harmonizome/) created by the Ma'ayan Laboratory of Computational Systems Biology, a comprehensive resource of knowledge about genes and proteins, to predict the possible phenotype of *LAMP*1 gene and the results were sorted according to the Z-score.

### Immune-infiltration analysis

We used the TIMER2.0 to estimate the levels of tumor-infiltrating immune cells in autism blood sample dataset GSE6575. The Pearson’s correlation coefficient between *LAMP*1 gene expression and levels of tumor-infiltrating immune cells was calculated using psych package (2.3.3) in R.

### Quantitative peptide analysis

Whole blood was collected from healthy children or children with ASD using EDTA anticoagulation tubes, and blood samples were centrifuged at 3000 rpm for 10 min at room temperature immediately after collection for plasma separation, and the upper layer of plasma was transferred to centrifuge tubes and immediately placed in – 80 °C for freezing. The plasma samples were selected from 3 groups of healthy children and 6 groups of children with ASD after pre-experimentation, and the indexes were measured according to the following procedure. The proteins were first quantified and then enzymatically digested, and all samples were labeled and quantified using TMT stable isotopes, while the samples were then separated by high performance liquid chromatography classification technique and finally subjected to LC–MS analysis. The secondary spectra obtained by mass spectrometry were searched for protein theoretical data to obtain validated spectra, and the specific peptides were identified by spectral analysis and quantitative protein analysis was performed.

### Statistical analysis

Statistical software SPSS 23.0 was used to analyze the experimental data, and GraphPad Prism 6.0 software was used for statistical graphing. Groups of continuous variables were compared by unpaired two-side Student's t test. Groups of categorical variables were compared by chi-square test. The correlation between *LAMP*1 expression and ASD-related scores was evaluated using the Pearson method. Differences were considered to be statistically significant for if *P.Value*s were < 0.05. Receiver operating characteristic (ROC) curves were generated with MedCalc19.6 software to evaluate their sensitivities and specificities in distinguishing ASD from healthy controls.

## Results

### Novel blood-based biomarkers dysregulated in ASD children

**Figure 1:**
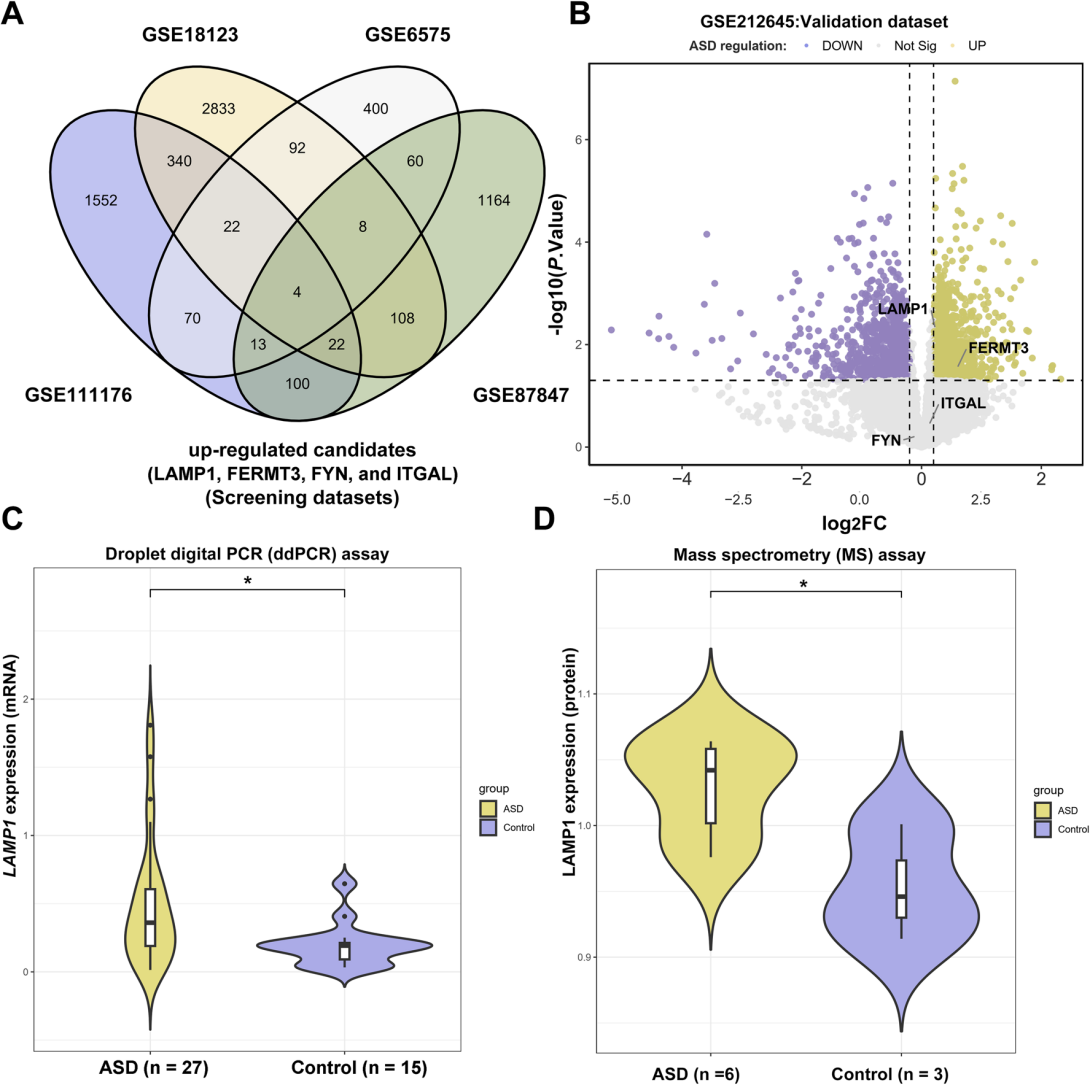
Identification of LAMP1 as a differentially expressed gene in blood samples of autistic children. We searched the GEO (Gene-expression-omnibus) database for microarrays of blood samples from autistic children and healthy controls and used the limma package to calculate the differential expressions. (**A**) The Venn diagram to show the four commonly up-regulated genes in autism subject blood samples from GSE6575, GSE18123, GSE87847 and GSE111176 datasets with a threshold of log_2_FC > 0.40 or < − 0.40 and *P.Value* < 0.05. (**B**) Volcano plot for the differentially expressed genes in GSE212645, an RNA sequencing of blood from sex- and age-matched discordant siblings, with yellow indicating up and purple indicating down-regulated expressions. (**C**) Violin plot for the *LAMP*1 mRNA expressed identified using the ddPCR assay based on the blood samples of the subjects from local hospital. (**D**) Violin plot for the LAMP1 protein expressed identified using protein MS assay based on the blood samples the subjects collected at local hospital. The Venn diagram and volcano/violin plots were generated using ggvenn (0.1.10) and ggplot2 (3.4.2) package respectively. The asterisk (*) indicates a statistically significant difference (*P.Value* < 0.05). *ddPCR* Droplet Digital PCR, *FC* Foldchange, *MS* mass spectrometry.

We looked for differential genes in four sets of blood samples (microarray and RNA-seq datasets) from children with ASD and normal control samples, including GSE6575 (Autism/General population: 35/12), GSE18123 (Autism/Control: 31/33), GSE111176 (Autism/Control: 91/56) and GSE87847 (Autism/typically developing: 21/24). We discovered four genes (*LAMP*1, *FERMT*3, *FYN*, and *ITGAL*) in ASD patients' blood samples that were significantly upregulated, with log_2_FC values greater than 0.40 and *P.Value*s less than 0.05 (Fig. 1A). We also further confirmed that *LAMP*1 (log_2_FC = 0.255, *P.Value* = 0.00679) and *FERMT*3 (log_2_FC = 0.538, *P.Value* = 0.0391) were significantly up-regulated in the GSE212645, an RNA sequencing dataset derived from blood samples of 27 pairs ASD and unaffected siblings (SIB) matched by sex and age (Fig. 1B). To further validate the dysregulated expression of *LAMP*1, blood samples were collected at local hospitals from 27 autistic children. The results of the Student’s t-test and the Chi-square test revealed that there was no difference in age or gender between the ASD and control group (*P.Value* > 0.05). We extracted RNA from all samples, converted it into cDNA using reverse transcription polymerase chain reaction (RT-PCR), and then performed droplet digital PCR (ddPCR) on it. After normalization with the expression of *GAPDH* (Glyceraldehyde 3-phosphate dehydrogenase), we showed that *LAMP*1 was up-regulated in blood samples from total ASD children (log_2_FC = 0.435, *P.Value* = 0.0219) (Fig. 1C). Meanwhile, we also collected plasma protein samples from children with ASD (n = 6) and age-matched controls (n = 3) and detected LAMP1 protein expression by quantitative protein analysis using mass spectrometry (MS), and also showed that LAMP1 protein expression was significantly upregulated in the plasma of ASD children (*P.Value* = 0.0288) (Fig. 1D).

### LAMP1 up-regulation in blood and brain samples in human autism cohorts and animal models

**Figure 2:**
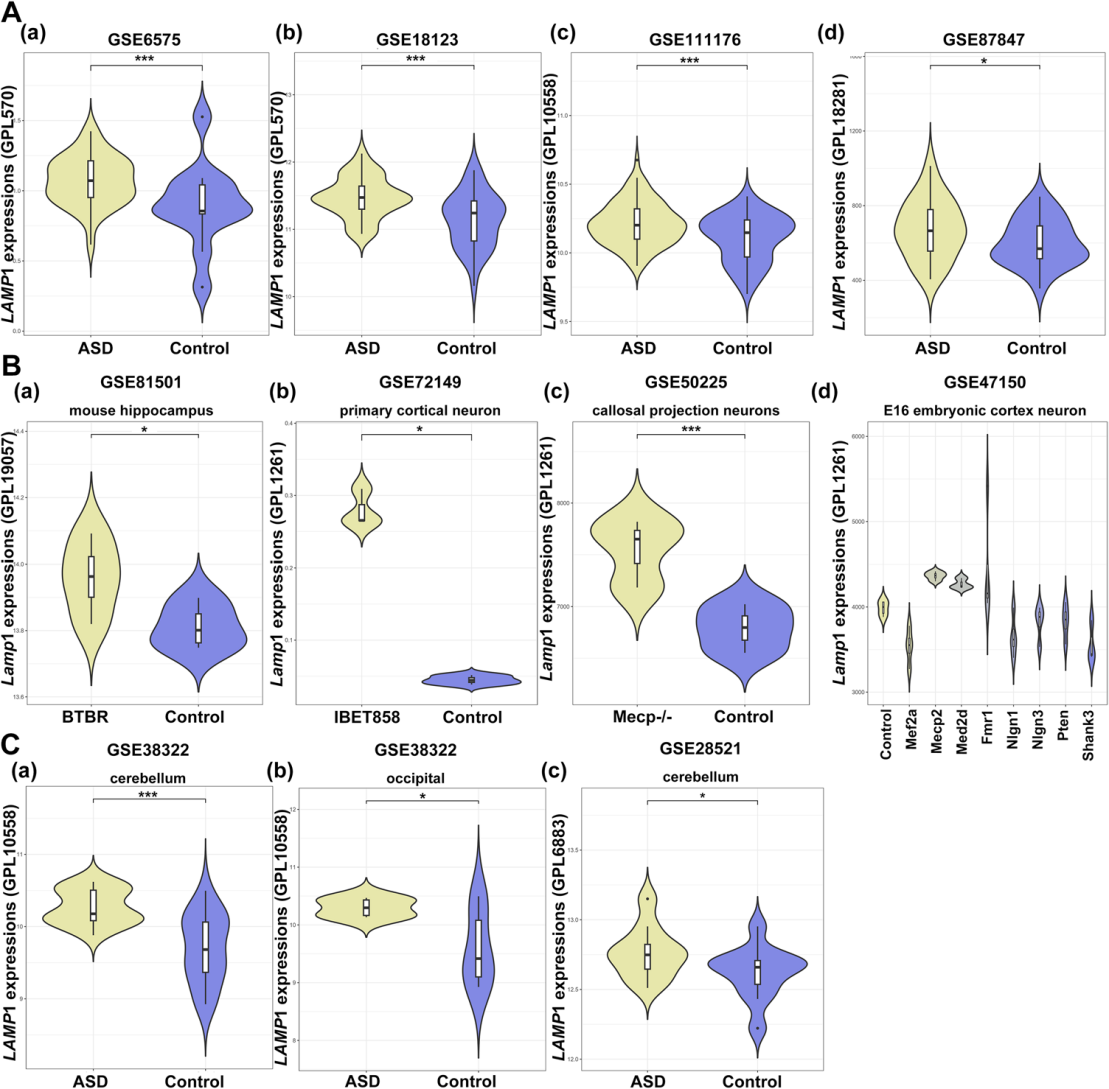
LAMP1 up-regulation in blood and brain samples of human autism cohorts and animal models. (**A**)The gene expression levels of *LAMP*1 in blood samples from ASD patients and healthy controls based on dataset GSE6575 (a), GSE18123 (b), GSE111176 (c) and GSE87847 (d). (**B**)The gene expression levels of *Lamp*1 in autistic mouse models. (a) *Lamp*1 expression in hippocampus tissues from autistic BTBR T^+^ ltrp3tf/J mice and normal brain tissues based on dataset GSE81501. (b) *Lamp*1 expression in mouse primary cortical neurons when treated with 1 µM IBET858 for 12 h in Mecp^−/−^ callosal projection neurons according to dataset GSE72149. (c) *Lamp*1 expression in callosal projection neurons from Mecp2-null mice in dataset GSE50225. (d) *Lamp*1 expression in the E16 primary cortical neuron cultures after transduction with shRNA constructs of ASD-implicated genes (ACGs): *Mecp*2, *Mef*2a, *Mef*2d, *Fmr*1, *Nlgn*1, *Nlgn*3, *Pten*, and *Shank*3 in dataset GSE47150. (**C**)The gene expression levels of *LAMP1* in brain tissues from ASD patients and healthy controls. (a, b) The gene expression levels of *LAMP*1 in cerebellum and occipital tissues from ASD patients and healthy controls based on dataset GSE38322. (c) The gene expression levels of *LAMP*1 in postmortem cerebellum tissue from ASD and control groups in dataset GSE28521. The asterisk indicates the level of statistically significance (*P.Value < 0.05 and ***P.Value < 0.01).

We discovered that *LAMP*1 gene expression levels were significantly upregulated in ASD blood sample datasets, including GSE6575 (log_2_FC = 0.628, *P.Value* = 0.0146), GSE18123 (log_2_FC = 0.671, *P.Value* = 0.000687), GSE111176 (log_2_FC = 0.631, *P.Value* = 0.000213) and GSE87847 (log_2_FC = 0.483, *P.Value* = 0.0367) (Fig. 2A). Further, in several animal models of autism, *Lamp*1 expression was found to be significantly upregulated in brain tissues or neurons (Fig. 2B). In dataset GSE81501, *Lamp*1 expression was higher in the BTBR mouse hippocampus than in the C57Bl6/J controls (5 months old, n = 4 mice per group) (log_2_FC = 1.472, *P.Value* = 0.0492). In GSE72149, elevated expression of *Lamp*1 was found in the primary mouse cortical neurons (log_2_FC = 4.098, *P.Value* = 0.0176) after 12 h inhibition of the bromodomain and extra-terminal domain containing transcriptional regulators (BETs), epigenetic drivers of an ASD-like disorder in mice. Interestingly, BDNF (Brain-derived neurotrophic factor) can suppress the up-regulated expression of *Lamp*1 induced by BET inhibition (log_2_FC = -0.303, *P.Value* = 2.93e-3). *Lamp*1 expression was found to be higher in callosal projection neurons from Mecp2-null mice in dataset GSE50225 (log_2_FC = -0.303, *P.Value* = 2.93e − 3). Mutations in the transcriptional regulator Mecp2 cause the X-linked ASD Rett syndrome (RTT). Furthermore, in dataset GSE47150, *Lamp*1 expression was increased in the E16 primary cortical neuron cultures after transduction with shRNA constructs of ASD-implicated genes (ACGs): *Mecp*2 (logfc = 9.571, *P.Value* = 0.0183), *Mef*2a and *Mef*2d, but not *Fmr*1, *Nlgn*1, *Nlgn*3, *Pten*, and *Shank*3 (*P.Value* > 0.05). Furthermore, we validated *LAMP*1 gene expression from additional ASD-related datasets in GEO (Gene-expression omnibus) database to further investigate the prevalence of aberrant *LAMP*1 expression. *LAMP*1 mRNA was found to be upregulated in cerebellum samples from ASD patients (Fig. 2C). In brain tissue microarray data GSE38322, we found that *LAMP*1 was significantly upregulated in both the cerebellum (log_2_FC = 0.508, *P.Value* = 2.80e − 4) and occipital tissues (log_2_FC = 0.707, *P.Value* = 0.0437) of ASD patients when compared to normal group. In dataset of GSE28521, *LAMP*1 was significantly increased in postmortem cerebellum tissues (log_2_FC = 0.495, *P.Value* = 0.0391), but has no difference between ASD patients and controls in the prefrontal cortex (*P.Value* > 0.05), as well as temporal lobes but not in frontal cortex (not shown).

### Function analysis suggesting the neuron and immune-related roles of LAMP1

**Figure 3:**
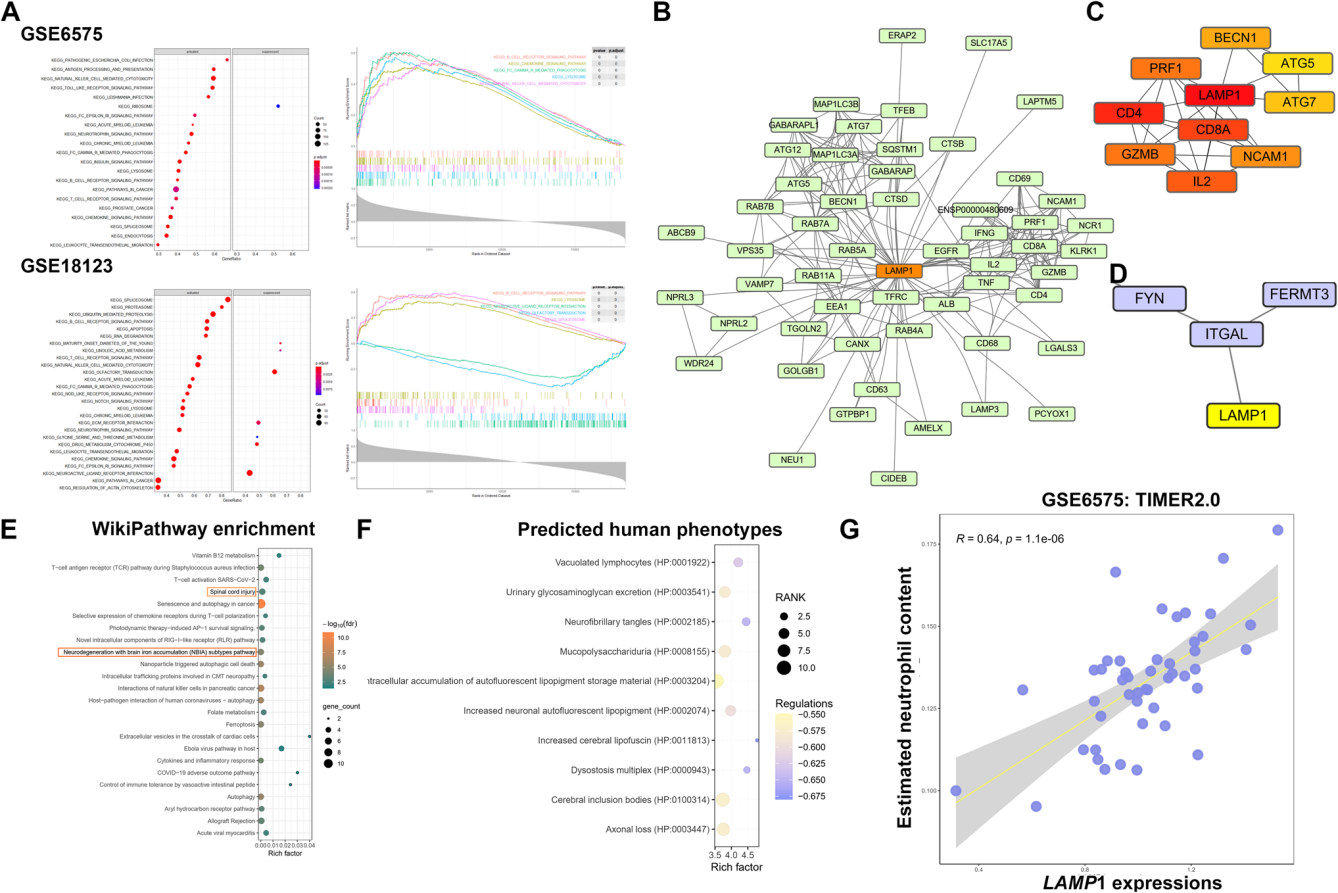
LAMP1 protein–protein interaction network reveals its role in immune regulation. (**A**) KEGG-GSEA enrichment analysis of *LAMP*1 correlated genes on GSE6575 (upper) and GSE18123 (lower) datasets. (**B**) The protein–protein interacting (PPI) network of LAMP1 was obtained from STRING (string-db.org) and visualized using Cytoscape software (3.8.4). (**C**) The top 10 hub-gene was calculated using cytoHubba plugin in software using Maximal Clique Centrality (MCC) algorithm. (**D**) PPI network connectivity for LAMP1 and DEGs genes identified as up-regulated in ASD groups constructed by STRING (https://string-db.org/). (**E**) The WikiPathway enrichment analysis according to PPIs of the STRING enrichment results. (**F**) Human phenotype prediction of *LAMP*1 using Harmonizome database indicating as Z-score. (**G**) Immune cell enrichment analysis of GSE6575 using TIMER2.0 and that correlation assay between *LAMP*1 mRNA expression associations with neutrophil enrichment content was performed. KEGG: Kyoto Encyclopedia of Genes and Genomes.

The GSEA (gene set enrichment analysis) enrichment analysis was first performed in two autism datasets, GSE6575 and GSE18123, using the KEGG (Kyoto Encyclopedia of Genes and Genomes) gene set. The findings show that *LAMP*1-related genes were significantly enriched in the functional pathways of T cells and NK cells in both sets of data, such as T cell receptor signaling pathway (hsa04660) and Natural killer cell mediated cytotoxicity (hsa04650) (Fig. 3A). The protein–protein interaction network around LAMP1 was constructed using STRING. We obtained a network of 55 nodes with 239 edges, with an average node degree of 8.69, an average local clustering coefficient of 0.791, and an expected number of edges of 74, with a PPI enrichment *P.Value* of less than 1.0e − 16 (Fig. 3B). The maximal clique centrality (MCC) algorithm is used to identify the top 10 hub-gene in PPI networks (Fig. 3C). LAMP1 is an immune-associated protein, as its top ten hub binding patterns include CD4 (Cluster of Differentiation 4), CD8A (Cluster of Differentiation 8A), GZMB (Granzyme B), and IL2 (Interleukin 2), which were largely expressed in Natural Killer-T cells. Hub genes also have players in autophagy pathway, such as ATG5 (Autophagy related 5), ATG7 (Autophagy related 7) and BECN1 (Beclin1). This finding could imply that LAMP1 is involved in intracellular stress-induced autophagy while also regulating immune cell activity extracellularly. More notably, LAMP1 also interact with ITGAL, FYN and FERMT3 which were dysregulated in our blood biomarker screening results, suggesting that these proteins involved in focal adhesion and immune regulation may play a vital role in autism (Fig. 3D). According to the wikipathway enrichment results in STRING, LAMP1-associated genes were enriched in pathways such as neurodegeneration and spinal cord injury (Fig. 3E). On the other hand, we used the Harmonizome database to predict the phenotype of LAMP1, which was significantly enriched in neuron function such as Neurofibrillary tangles (HP:0002185), increased cerebral lipofuscin (HP:0011813), Axonal loss (HP:0003447), Cerebral inclusion bodies (HP:0100314) and increased neuronal autofluorescent lipopigment (HP:0002074) (Fig. 3F). We performed immune cell enrichment analysis of GSE6575 using TIMER2.0 and we found that LAMP1 expression significantly positively correlated with neutrophil enrichment content (*R* = 0.64, *P.Value* = 1.1e − 06) (Fig. 3G).

### The correlations between LAMP1 expressions and clinical ASD rating scale

**Figure 4:**
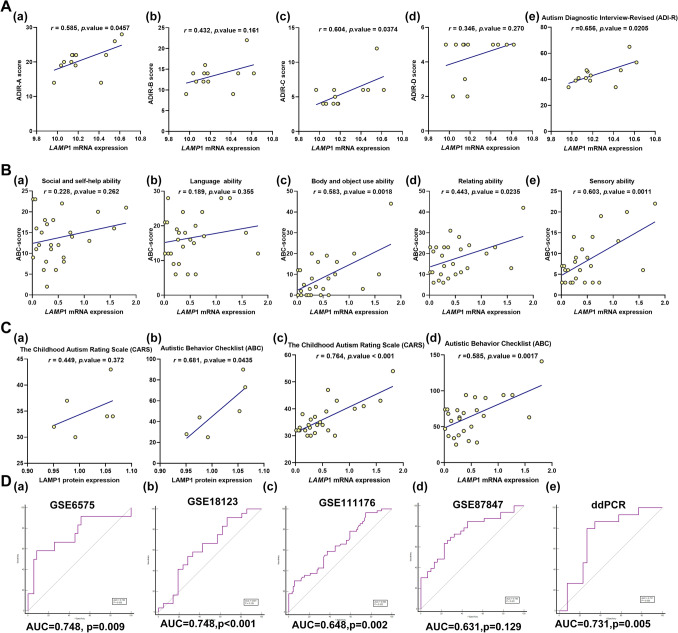
The correlations between LAMP1 expressions and clinical ASD rating scales. (**A**) The correlations between LAMP1 gene expressions in cerebella and Autism Diagnostic Interview-Revised (ADIR) total or ADIR domain scores based on dataset GSE38322. ADIR-A: Social interaction impairments domain; ADIR-B: Communication and language impairments domain; ADIR-C: Repetitive and stereotyped behaviors domain; ADIR-D: Symptom onset before 36 months of age; ADIR total: Sum of scales A through D. (**B**) The correlations between *LAMP*1 gene expressions in blood samples and ABC (Autism Behavior Checklist) including social and self-help ability (a), language ability (b), body and object ability (c), relating ability (d) and sensory ability (e). (**C**) The correlations between LAMP1 protein and mRNA expressions in blood samples and Childhood Autism Rating Scale (CARS) scores and ABC total from local autistic children. (**D**) Receiver operating characteristic (ROC) curves of LAMP1 for prediction of ASD and healthy controls based on dataset GSE6575 (a), GSE18123 (b), GSE111176 (c), GSE87847 (d) and droplet digital PCR results from local autistic children (e). Sensitivity (true-positive rate) and 1-specificity (false-positive rate); *AUC* area under curve.

Further we explored the correlation between LAMP1 expression and clinical features. The blood sample expression of *LAMP*1 was correlated using CARS (Childhood autism rating scale) and ABC (Autism Behavior Checklist) scores. We analyzed the correlation between *LAMP*1 gene expressions in cerebella and Autism Diagnostic Interview-Revised (ADI-R) total or ADI-R domain scores based on dataset GSE38322 (Fig. 4A). In the ASD group, *LAMP*1 expressions significantly positive correlated with ADI-R social interaction impairments domain scores and ADI-R repetitive and stereotyped behaviors domain scores respectively, but no significantly associated with ADI-R communication and language impairments domain scores and ADI-R symptom onset before 36 months of ages cores. Furthermore, in local autistic children, *LAMP*1 gene expressions in blood samples significantly positive correlated with ABC body and object use ability domain scores(*r* = 0.583, *P.Value* = 0.0018), ABC relating ability domain scores(*r* = 0.443, *P.Value* = 0.0235) and ABC sensory ability domain scores(*r* = 0.603, *P.Value* = 0.0011) respectively, but no significantly associated with ABC social and self-help ability domain scores(*r* = 0.228, *P.Value* = 0.263) and ABC language ability domain scores(r = 0.189, *P.Value* = 0.355)(Fig. 4B). LAMP1 protein expression was found to be significantly related to total ABC scores (*r* = 0.681, *P.Value* = 0.0435), but not to Childhood Autism Rating Scale (CARS) scores (r = 0.449, *P.Value* = 0.372) (Fig. 4C: a,b). Furthermore, *LAMP*1 gene expression in blood samples was found to be significantly correlated with CARS scores from local autistic children (Fig. 4C: c,d). To examine the diagnostic efficacy of the candidate biomarker LAMP1, the ROC curves of LAMP1 in blood samples for prediction of ASD and healthy controls were drawn from dataset GSE6575, GSE18123, GSE111176, GSE87847 and local autistic children based on droplet digital PCR results. ROC analysis showed that the area under the ROC curve (AUC) of GSE6575, GSE18123, GSE111176 and GSE87847 reached 74.8%, 74.8%, 64.8%, 63.1% respectively, which could be considered to have a good degree of differentiation (Figs. 4D). In blood samples data from local autistic children based on droplet digital PCR results, the AUC of LAMP1 to significantly distinguish between the normal group and the ASD group was 77.1% (Fig. 4D), showed that LAMP1 might be a good marker for improving the accuracy of prognostic ASD prediction.

### LAMP1 genomic methylation level altered in ASD blood samples

**Figure 5:**
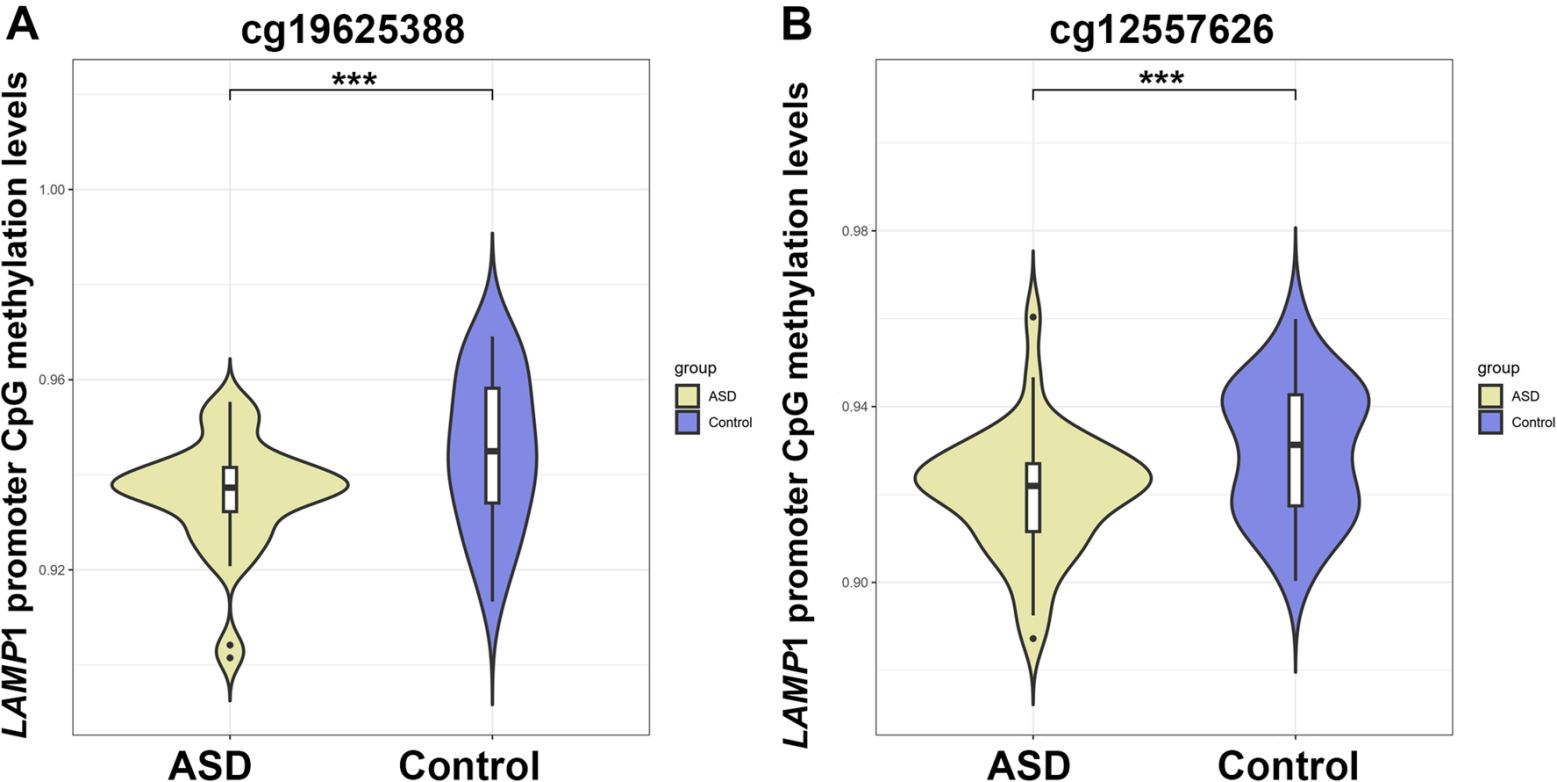
LAMP1 methylation altered in ASD patients’ blood samples. We investigated the differential methylation levels around the *LAMP*1 genomic region using GSE109905, a genome-wide DNA methylation profile of ASD (n = 38) and controls (n = 31) utilizing peripheral blood samples based on the platform GPL13534, the Illumina Infinium HumanMethylation450 (450K) BeadChip array. The Beta Mixture Quantile Dilation (BMIQ) approach was used to standardize the beta values. The differentially methylated sites (DMPs) were examined using the limma program. To measure methylation levels, the ratio of intensities between methylated and unmethylated alleles (beta-values) is utilized. Two CpG sites, cg19625388 (P.Value = 7.28e − 03) and cg12557626 (P.Value = 7.81e − 03) with most significant differential methylation levels was shown (**A**) and (**B**).

Methylation levels are important for the regulation of gene expression. The differentially methylated CpG sites between ASD patients and controls around the genomic region of the *LAMP*1 was analyzed in blood samples from 38 ASD patients and 31 controls in the dataset GSE109905. Among 34 CpG sites around human *LAMP*1 genomic region, methylation levels of 10 sites (cg19625388, cg12557626, cg00994984, cg23439141, cg07161140, cg25408055, cg13804476, cg06844489, cg10997500, cg01330448) were significantly altered (*P*.*Value* < 0.05). The most significant altered methylation levels of CpG sites between ASD patients and controls were cg19625388 (*P*.*Value* = 7.28e − 03) and cg12557626 (*P*.*Value* = 7.81e − 03), both located around the 3’UTR of *LAMP*1 genomic region (Fig. 5).

## Discussions

Early screening and early diagnosis of ASD play a key role in the prognosis of this disease^11^. According to the results of correlation analysis, *LAMP*1 gene expression was significantly correlated with ADI-R, CARS and ABC scores of ASD patients. In the correlation analysis of domain scores, we were able to further see that the gene expression of *LAMP*1 was strongly correlated with the body movement’s ability of ASD patients in both ADI-R scores and ABC scores, but not with language ability and social skills (Fig. 4A and B). The diagnostic value of LAMP1 in ASD was verified using ROC analysis using both published datasets and our own data (Fig. 4D).

There is increasing evidence that the cerebellum is closely associated with ASD^12, 13^. Abnormalities in the cerebellum of autistic patients were found in some early pathological autopsy findings, and they suggested that the degree of pathological alterations was highly correlated with cognitive dysfunction, social impairment and repetitive-like motor behavior in autistic patients^14^. The cerebellum is likely to be an important brain region for the development of motor deficits and other abnormal behaviors in the pathology of autism. Although current pathological studies of ASD have focused on the prefrontal cortex and limbic system, and the cerebellum has been stereotypically involved only in the regulation of motor coordination^15, 16^. The specific upregulation of LAMP1 expression in the cerebellum (Fig. 2C) suggests that LAMP1 may influence the neurodevelopment of the organism by regulating cerebellar dysfunction.

Immune dysregulation does occur frequently in ASD patients, and it is likely that this dysregulation is related to the pathogenesis and/or severity of ASD^1, 17^. There is growing evidence that immune system and abnormal immune function (including inflammation, cytokine dysregulation, and anti-brain autoantibodies) affects the brain during development and has been implicated as a key factor in the development and maintenance of ASD^10, 18^. The activity of T cell and NK cell subsets may be altered in ASD patients and their response to stimuli may be diminished. Disruption of normal cytokine levels has an important role in the development of ASD.

LAMP1, as a type I transmembrane protein, is not only responsible for maintaining the structural integrity of lysosomes and pH stability within lysosomes, but also participates in various intracellular physiological processes, such as regulation of lysosomal cytosolic exocytosis^19^ and cholesterol transport^20^. There is growing evidence that LAMP1 is involved in immune dysregulation. Previous study has demonstrated that LAMP1 is involved in protecting NK cells from degranulation-associated damage^7^. Also, LAMP1 might be a future therapeutic target to address cytotoxic T-cells, as a critical role for CD8 + T-cell activation in particular in systemic lupus erythematosus^8^. Recent research show that LAMP1 + TRAIL + astrocytes limit CNS inflammation by inducing T cell apoptosis, and that this astrocyte subset is maintained by meningeal IFNγ + NK cells that are licensed by the microbiome^21^. LAMP1 has been reported to be associated with the development of several neurological diseases, such as Alzheimer’s disease^22^, neuroinflammatory^23^ and Niemann-Pick disease^24^. Previous study found that LAMP1 spots increased in Mucopolysaccharidosis (MPS) III cells, a severe inborn metabolic error caused by mutations of the sulfamidase gene (SGSH), a lysosomal enzyme that participates in the metabolism of the glycosaminoglycan (GAG), heparan sulfate (HS), associated with neurodegeneration and dementia^25^. Furthermore, Huang found LAMP1 was involved in the gene regulatory networks associated with ASD based on miRNA expression in China^26^. Here, our study found that LAMP1 forms an interaction network with NKT cell marker factors. It might be possible that the abnormal upregulation of LAMP1 expression may be involved in the ASD process by altering the activity of NK/T cells, leading to an imbalance of immune regulation.

In summary, we identified LAMP1 could be a molecular biomarker in the development of ASD. The advantage of this study is that we fully evaluated diagnostic value of LAMP1 as it has been validated on local ASD children and multiple independent datasets, and might imply that LAMP1 with a potential function in ASD. The biological functions of the obtained pivotal gene need to be further validated by means of in vivo and ex vivo experiments and larger clinical sample size testing in the future.

### Supplementary Information

Supplementary Tables.

## Data Availability

The datasets analyzed during the current study are available in the Gene Expression Omnibus (GEO) (https://www.ncbi.nlm.nih.gov/geo), including GSE6575, GSE18123, GSE87847, GSE111176, GSE212645, GSE81501, GSE72149, GSE50225, GSE47150, GSE38322, GSE28521 and GSE109905.
