# Supplementary material for: LAMP1 as a novel molecular biomarker to predict the prognosis of the children with autism spectrum disorder using bioinformatics approaches
Source: Sci Rep. 2023 Aug 28;13:14040. doi: 10.1038/s41598-023-40617-4 (PMC10462740; doi:10.1038/s41598-023-40617-4)
Supplement: Supplementary file 1 — Supplementary Tables. [file 41598_2023_40617_MOESM1_ESM.docx]

Supplemental table 1. Descriptive statistics for the ASD and Normal control groups.

| **Characteristic** | **ASD subjects** | **Normal control** | ***p*** |
| --- | --- | --- | --- |
| n | 27 | 15 |  |
| Gender, n (%) |  |  | 0.225 |
| Female | 3 (11.1%) | 4 (26.7%) |  |
| Male | 24 (88.9%) | 11 (73.3%) |  |
| Age in years |  |  |  |
| Mean (SD) | 3.85 (0.77) | 4.40 (1.12) | 0.106 |
| Range | 3 - 5 | 3 - 7 |  |
| CARS Score |  |  |  |
| Mean (SD) | 35.78 (5.793) |  |  |
| Range | 30 - 54 |  |  |
| ABC Score |  |  |  |
| Total Score Mean (SD) | 62.81 (27.54) |  |  |
| Total Score Range | 18 - 141 |  |  |
| Body and Object Use Ability  Domain Score Mean (SD) | 8.11 (9.88) |  |  |
| Body and Object Use Ability  Domain Score Range | 0 - 44 |  |  |
| Relating Ability  Domain Score Mean (SD) | 17.07 (8.92) |  |  |
| Relating Ability  Domain Score Range | 4 - 42 |  |  |
| Sensory Ability  Domain Score Mean (SD) | 7.96 (5.71) |  |  |
| Sensory Ability  Domain Score Range | 0 - 22 |  |  |
| Social and Self-help Ability  Domain Score Mean (SD) | 13.26 (6.02) |  |  |
| Social and Self-help Ability  Domain Score Range | 1 - 23 |  |  |
| Language Ability  Domain Scores Mean (SD) | 16.37 (6.56) |  |  |
| Language Ability  Domain Scores Range | 6 - 28 |  |  |

Supplemental table 2. The primer sequences used for ddPCR.

| **Gene** | **Forward primer sequence (5′→3′)** | **Reverse primer sequence (5′→3′)** |
| --- | --- | --- |
| LAMP1 | TCTCAGTGAACTACGACACCA | AGTGTATGTCCTCTTCCAAAAGC |
| GAPDH | CTGGGCTACACTGAGCACC | AAGTGGTCGTTGAGGGCAATG |
